# Drug resistance associated with loss of p53 involves extensive alterations in microtubule composition and dynamics

**DOI:** 10.1038/sj.bjc.6600960

**Published:** 2003-05-27

**Authors:** C M Galmarini, K Kamath, A Vanier-Viornery, V Hervieu, E Peiller, N Falette, A Puisieux, M Ann Jordan, C Dumontet

**Affiliations:** 1 INSERM 590-Laboratoire de Cytologie Analytique, Faculté de Médécine Rockefeller, Lyon 69373, France; 2 Department of Molecular, Cellular and Developmental Biology, University of California at Santa Barbara, CA 93106, USA

**Keywords:** protein p53, cytoskeleton, microtubules, drug resistance, antineoplastic agents

## Abstract

In the present study, we compared the dynamics and composition of microtubules in cell lines derived from the human breast adenocarcinoma MCF-7 containing either the wild-type *p53* (wt-p53; MN1) or a dominant-negative variant of *p53* gene (mut-p53; MDD2). Mut-p53 cells were significantly resistant to the cytotoxicity of the microtubule-targeted drugs (vinca alkaloids and taxanes), as compared with wt-p53 cells. Studies by high-resolution time-lapse fluorescence microscopy in living cells indicated that the dynamics of microtubules of mut-p53 cells were altered in complex ways and were significantly increased as compared with microtubules in wt-p53 cells. The percentage of time microtubules spent in growing and shortening phases increased significantly, their catastrophe frequency increased, and their overall dynamicity increased by 33%. In contrast, their shortening rate and the mean length shortened decreased. Cells containing mut-p53 displayed increased polymerisation of tubulin, increased protein levels of the class IV *β*-tubulin isotype, STOP and survivin, and reduced protein levels of class II *β*-tubulin isotype, MAP4 and FHIT. We conclude that p53 protein may contribute to the regulation of microtubule composition and function, and that alterations in p53 function may generate complex microtubule-associated mechanisms of resistance to tubulin-binding agents.

The effect of p53 tumour suppressor gene status on sensitivity to microtubule-targeted drugs has been a subject of controversy, and may be different whether one considers normal or neoplastic cells (Wahl *et al*, 1996; McGill and Fisher, 1997; Brown and Wouters, 1999). In cancer cells, alterations of the *p53* tumour suppressor gene confer resistance to microtubule-targeted drugs (Wu and El-Diery, 1996; Galmarini *et al*, 2001). We recently reported that chemoresistance present in *p53*-deficient cells might be attributed to defective apoptotic pathways (Galmarini *et al*, 2001). However, *p53* also associates with microtubules *in vitro* and *in vivo* (Giannakakou *et al*, 2000b), and has been reported to regulate negatively the putative microtubule-stabilising protein microtubule-associated protein 4 (MAP4), the microtubule-related inhibitor of apoptosis survivin (Mirza *et al*, 2002), and the microtubule-destabilising protein stathmin/OP18 (Johnsen *et al*, 2000; Murphy *et al*, 1999). Whether these interactions of *p53* with the microtubule network directly affect microtubule composition and dynamics thus favouring drug resistance is not known.

The dynamic behaviour of microtubules includes dynamic instability, in which the ends of the microtubule undergo frequent episodes of slow growing and rapid shortening, as well as treadmilling, which is the net addition of tubulin at one end of a microtubule and net loss at the other end. Both phenomena are important for cell cycle progress as they play critical roles in the assembly and function of the mitotic spindle and cytokinesis (Margolis and Wilson, 1998; Rodionov *et al*, 1999).

Specifically, highly dynamic microtubules are required for the proper attachment of chromosomes to the spindle and for the complex movements of the chromosomes (called ‘congression’), including their proper alignment at metaphase and chromosome separation at anaphase. Failure of the microtubules to capture all the chromosomes at prometaphase, as well as the absence of tension on the chromosomal kinetochores during metaphase, leads to mitotic block (Rudner and Murray, 1996) and apoptosis (Jordan *et al*, 1996).

Recent studies have indicated that microtubule-targeted drugs (e.g. vinblastine, paclitaxel) block mitosis at the metaphase/anaphase transition by suppressing dynamic instability and treadmilling (Yvon *et al*, 1999; Goncalves *et al*, 2001). Moreover, changes in the composition and dynamics of microtubules appear to contribute to specific resistance to microtubule-targeted drugs (reviewed in Dumontet and Sikic, 1999).

In this study, we used cell lines derived from the human breast adenocarcinoma MCF-7 containing either wild type (*wt*)*-p53* or a dominant-negative variant of the *p53* gene (previously shown to be resistant to microtubule-targeted drugs, Galmarini *et al*, 2001), as a model to investigate the possible relation between p53 expression, dynamics and composition of microtubules and drug resistance.

## MATERIAL AND METHODS

### Reagents

Antibodies against acetylated tubulin (6-11B-1), tyrosinated tubulin (Tub-1A2), *β*-tubulin (Tub 2. 1), *γ*-tubulin, tau (Tau-2), MAP2 (HM-2), MAP1b/MAP5 (AA6) and *β*-actin (AC-15) were purchased from Sigma (St Quentin Fallavier, France); antibodies against class IV *β*-tubulin isotype were purchased from Biogenex (San Ramon, CA, USA). Antibodies against class II (7B9 clone) and class III *β*-tubulin isotype (TUJ1 clone) were generously provided by Anthony Frankfurter (University of Virginia, Charlottesville, VA, USA), against STOP (23.5) and glutamylated tubulin (L7) by Didier Job (Grenoble, France); against MAP4 by Chloé Bulinski (New York). Peroxidase-conjugated secondary antibodies were from Covalab (Oullins, France), enhanced chemiluminiscence Western blot detection reagents from Amersham ECL system (Amersham Corp., Buckinghamshire, UK). Vinblastine was provided by Lilly Laboratories (Saint Cloud, France), paclitaxel by Bristol-Myers Squibb (Paris, France).

### Cell lines

The wt-p53-containing MN-1 cell line and the MDD2 cell line, derived from the human breast carcinoma cell line MCF-7, were provided by Moshe Oren (Weizmann Institute of Science, Israel). The MDD2 subline is an isogenic variant obtained from MCF-7 by transfection with a dominant-negative mutant (pCMV-DD-p53) coding for a p53 miniprotein containing the first 11 residues and residues 302–390 of mouse p53 (Shaulian *et al*, 1992). MN1 cells were obtained by transfection of the MCF-7 line with the empty plasmid. HCT116 p53−/− is a cell line derived from colorectal carcinoma in which two promoterless targeting vectors were used to disrupt sequentially the two p53 alleles (Bunz *et al*, 1998). All cell lines were cultured as previously described (Galmarini *et al*, 2001).

We have previously shown that MDD2 cells displayed 31.5-fold resistance to paclitaxel (MN-1 IC_50_: 7.6 nM; MDD2 IC_50_: 240 nM) and 50-fold resistance to vinblastine (MN-1 IC_50_: 6.8 nM; MDD2 IC_50_: 340 nM) in comparison with MN-1 cells (Galmarini *et al*, 2001).

### Dynamics measurements

Microinjection of labelled tubulin, image acquisition and dynamic measurements were performed as previously described (Goncalves *et al*, 2001). Bovine brain tubulin was labelled with carboxyrhodamine succinimidyl ester (Molecular Probes, Inc., Cergy, France) according to Hyman (1991). Cells were seeded at low density 2–3 days prior to microinjection on gridded glass coverslips (Eppendorf Scientific, Inc., LEPECQ, France). Injections were performed on a Zeiss Telaval 31 inverted microscope using a × 40 phase objective lens, and an Eppendorf Transjector 5246 and Injectman microinjection system. Following injections, cells were incubated for 4–6 h prior to analysis to allow for incorporation of rhodamine-tubulin into microtubules. Microinjected cells were visualised on a Nikon Eclipse E800 fluorescence microscope using a 1.4 N.A.100 × plan apochromatic lens at 36.5±0.5°C. The positions of the plus-ends of individual microtubules in the peripheral lamellar region of cells were tracked over time using the MetaMorph track points program (Universal Imaging, Westchester, PA, USA), transferred to a Microsoft Excel spreadsheet and graphed as position *vs* time to generate a ‘life-history plot’ for each microtubule using Real Time Measurement software (a kind gift from E Gliksman and E Salmon). From these graphs, growth and shortening rates and durations were derived by regression analysis. A difference of >0.5 *μ*m between any two consecutive points was considered as a growth or shortening event.

Transitions into microtubule depolymerisation or shortening are termed catastrophes, and transitions from shortening to growth or pause are called rescue. The catastrophe frequencies per unit time and per length grown were calculated by dividing the number of transitions from growth and pause to shortening by either the sum of the time in growth and pause or the sum of the distance grown, respectively. Similarly, time and length-based rescue frequencies were determined by dividing the number of transitions from shortening to growth or pause by the time spent or the distance shortened. Dynamicity is a measure of total tubulin exchange at the end of a microtubule and was calculated by dividing the sum of the total length grown and shortened by the lifespan of the microtubule.

### Reverse transcriptase–polymerase chain reaction

The level of mRNA expression of *β*-tubulin, *γ*-tubulin and STOP was assessed by semiquantitative reverse transciptase–polymerase chain reaction (rt–PCR) in a Perkin Elmer 9600 thermal cycler as previously described (Dumontet *et al*, 1999). After standardisation using ribosomal 18S primers, the PCR products of the genes of interest were produced at 35 cycles (PCR profile: denaturation at 94°C for 10 s, annealing at 56°C for 3 s, and elongation at 72°C for 30 s). Primer sequences and the number of cycles were as follows: *β*-tubulin: forward: ATGAGGGAAATCGTG; reverse: AGTGGGTCAGCTGGAAGC; *γ*-tubulin: forward: AGTTGGCCAACTTCATCC; reverse: TGCCCCAGGAGATGTAGT; STOP: forward: GAGCAGCTCCTACAGGAATGA; reverse: TGAAGGGTTCDCTGTAGAGG. The PCR products were then separated by electro-phoresis on a 6% polyacrylamide gel and visualised by staining with ethidium bromide. All samples were analysed in three separate experiments.

### Western blots

Protein expression was determined by Western blot analysis in MN-1 and MDD2 cells at basal conditions as described previously (Galmarini *et al*, 2001). Horizontal scanning densitometry was performed on Western blots by acquisition into Adobe Photo Shop (Apple, Cupertino, CA, USA) and analysis by the Kodak Digital Science 1D image analysis software.

### Evaluation of total polymerised and soluble tubulin

Cells in pellets were lysed by resuspension in 100 *μ*l of low-salt buffer (20 mM Tris-HCl pH 6.8; 1 mM MgCl_2_; 2 mM EGTA) for 5 min at 37°C in the dark, then centrifuged at 14 000 r.p.m. for 10 min at room temperature. The resultant supernatants, containing the soluble tubulin fraction, were transferred to a separate centrifuge tube and kept on ice. The pellets, containing polymerised tubulin, were resuspended in Ling's buffer (10 mM Tris pH 7.5, 1.5 mM MgCl_2_ and 10 mM KCl) in a volume equal to that of the supernatants. Protein extracted from MN-1 cells (100 *μ*g) and from MDD2 cells (300 *μ*g) were loaded onto a 12% acrylamide–SDS gel, then processed for immunoblotting with a pan-*β*-tubulin antibody as described above. The results were expressed as the ratio of polymerised/soluble tubulin for each cell line.

### Luciferase assays

One million HCT116−/− p53−/− cells were cotransfected with 325 ng of the pGL-3-STOP-luc plasmid containing the entire STOP promoter coupled to luciferase (generously given by E Denarier, Grenoble, France) and 50 ng of the pCMV-wt-p53 plasmid containing the complete coding sequence of wt-p53, using lipofectine (GibcoBRL, Cergy, France). Cells were harvested in reporter lysis buffer (Promega, Charbonnierbs, France) 48 h after transfection, and luciferase activity was quantified with a luminometer. Representative results of triplicate measurements of duplicate experiments with mean and standard deviation are shown in the figures. As a positive control, HCT1116 p53−/− cells were cotransfected with the pGL-3-p21/Waf1-luc plasmid and the pCMV-wt-p53 plasmid. Samples were analysed in three separate experiments

## RESULTS

### Microtubule dynamics in living cells expressing wt or mutated p53

**Figure 1:**
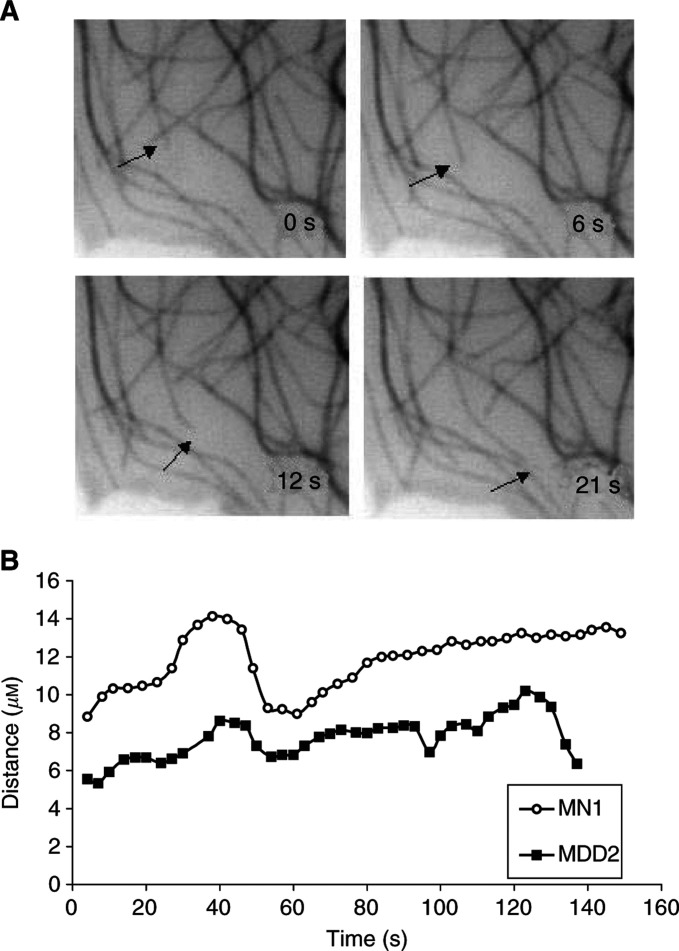
(**A**) Dynamic behaviour of microtubules in living MN1 cells. Arrows indicate a microtubule that undergoes a growing event. Time is indicated in seconds. (**B**) Life-history plots of single microtubule from living MN1 and MDD2 cells.

The dynamic behaviour of microtubules in MDD2 cells is illustrated in a time-lapse sequence (Figure 1A

). Microtubules alternated between phases of growing, shortening, and a pause state (a state of attenuated dynamic activity). The dynamic behaviour of single microtubule in living MN1 and MDD2 cells is graphed in Figure 1B in what are known as ‘life-history plots’, which shows the changes in length of single microtubules over time. The life-history plots of a number of microtubules were used to determine the parameters of dynamic instability (Tables 1

**Table 1:** Parameters of dynamic instability in MN1 and MDD2 cells

**Parameters**	**MN1 (wt-p53)**	**MDD2 (mut-p53)**	**Percentage of change^a^**
*Mean rate (μm min*^−1^*)*			
Growth	8.6±0.9^b^	8.9±0.8	+1
Shortening	29.1±2.8	21.7±1.7*	−25
			
*Mean duration (min)*			
Growth	0.29±0.03	0.32±0.04	+6
Shortening	0.18±0.02	0.16±0.01	0
Attenuation	0.51±0.04	0.35±0.03*	−29
			
*Mean length change/event (μm)*			
Growth	2.5	2.8	+12
Shortening	5.2	3.5	−33
			
*Percentage time spent*			
Growth	25.4	39.7	+56
Shortening	12.8	17.1	+34
Attenuation	61.8	43.2	−30
Dynamicity (*μ*m min^−1^)	5.9	7.9	+33

* *P*<0.02; estimates of significance by Student's *t*-test.

a The percentage difference relative to the corresponding parameter between MDD2 cells and MN1 cells. Parameters of dynamic stability were determined by measurement of at least 31 microtubules from seven to 12 cells for each cell type.

b s.e.m.

and 2

**Table 2:** Transition frequencies in MN1 and MDD2 cells

**Transition frequency**	**MN1 (wt-p53)**	**MDD2 (mut-p53)**	**Percentage of change^a^**
*Events min*^−*1*^			
Catastrophe	0.84±0.12	1.2±0.2	+43%
Rescue	4.2±0.7	4.4±0.6	+5%
			
*Events μm*^−*1*^			
Catastrophe	0.42±0.06	0.40±0.05	−5%
Rescue	0.15±0.03	0.22±0.03	+47%

a The percentage difference in MDD2 cells relative to the corresponding behavioural parameter in MN1 cells.

).

The dynamics of microtubules in mut-p53 cells were altered in significant and complex ways from those in the wt-p53 cells (Tables 1 and 2 and Figure 1). Changes in several parameters of microtubule dynamics indicated that the dynamics of microtubules in mut-p53 cell microtubules were significantly increased. The microtubules of mut-p53 cells spent less time in pause (62% in wt-p53 *vs* 43% in mut-p53 cells), with a correspondingly greater fraction of time spent in growing or shortening. Their catastrophe frequency per minute was increased significantly, by 43% (Table 2), which is consistent with and may account for the 29% reduction in the duration of pause events. The mean length shortened was reduced by 33% (from 5.2 to 3.5 *μ*m). The frequency of rescue based on length shortened was increased by 47% (Table 2), which is consistent with and may account for the decrease in shortening length. Overall, their dynamicity increased by 33%. Interestingly, in one way their dynamics were clearly suppressed, that is, their shortening rate was reduced by 25% (from 29.1±2.8 to 21.7±1.7 *μ*m min^−1^). The net result of these changes was that microtubules in mut-p53 cells underwent brief episodes of short-length changes. That is, relatively frequent catastrophes occurred during which they lost relatively little polymer length as a result of the slowed shortening rates. These changes are likely to deter significantly the proper formation of a mitotic spindle.

### Analysis of microtubule protein content at basal condition

**Figure 2:**
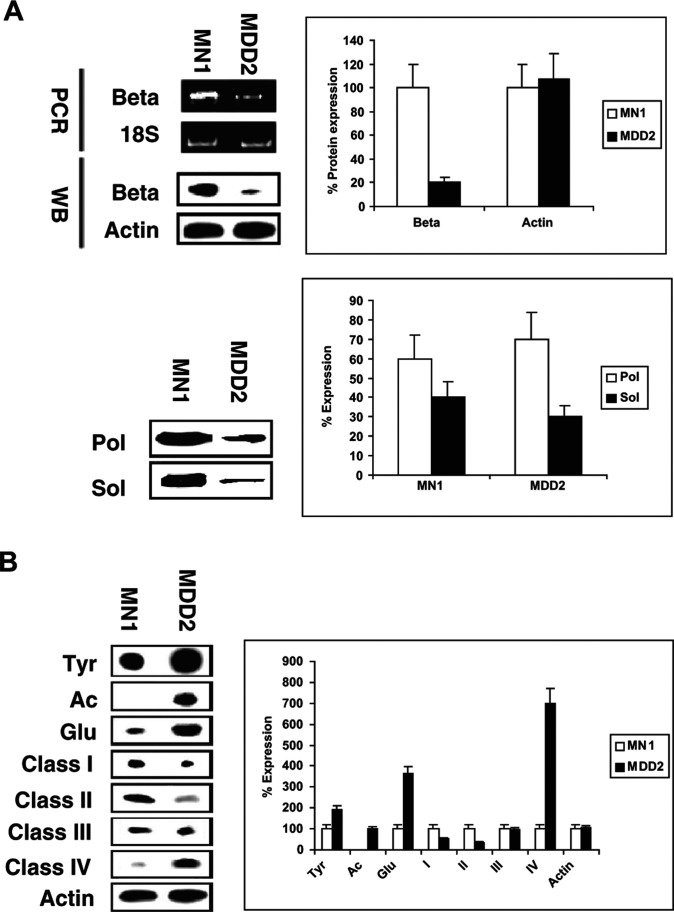
Comparisons of microtubule components between MN1 (wt-p53) and MDD2 (mut-p53) cells by Western blot at baseline. (**A**) Beta: *β*-tubulin; Actin: *β*-actin; Pol: polymerised *β*-tubulin; Sol: soluble *β*-tubulin; for Pol and Sol, the results shown are expressed as percentage of total tubulin. (**B**) Tyr: tyrosinated *α*-tubulin; Ac: acetylated *α*-tubulin; Glu: glutamylated *α*-tubulin; I: class I *β*-tubulin; II: class II *β*-tubulin; III: class III *β*-tubulin; IV: class IV *β*-tubulin; Actin: *β*-actin. These figures represent one of the two (A) or three (B) experiments performed.

By Western blotting of total cell extracts, we found that the total *β*-tubulin protein content was decreased in mut-p53 cells (Figure 2A

). Analysis of total *β*-tubulin mRNA levels by rt–PCR using pan-*β* primers also showed higher levels in wt-p53 cells (Figure 2A), confirming that *β*-tubulin expression was downregulated in the mut-p53 cells and suggesting that regulation of tubulin levels was transcriptional.

The ratio of polymerised tubulin to soluble tubulin was determined by Western blotting and densitometry. We found that it required larger amounts of protein from MDD2 cells to obtain sufficient signal to determine the ratio of polymer/soluble tubulin than from MN1 cells; the reasons for this difference are not known, but it appears that tubulin is less abundant overall in the mut-p53 cells than in the wt cells. The results also suggest that the ratio of polymerised tubulin to soluble tubulin was slightly higher in mut-p53 (2.3) than in wt-p53 cells (1.5) (Figure 2A).

In the MDD2 cells, the amount of Class IV *β*-tubulin was greatly increased, by seven-fold (Figure 2B), whereas Classes I and II were reduced and Class III was unchanged in comparison with wt-p53-containing MN-1 cells. Post-translational modifications of *α*-tubulin were different between mut-p53 and wt p53 cells with higher contents of tyrosinated, acetylated and glutamylated tubulin protein in the mut-p53 cells (Figure 2B).

**Figure 3:**
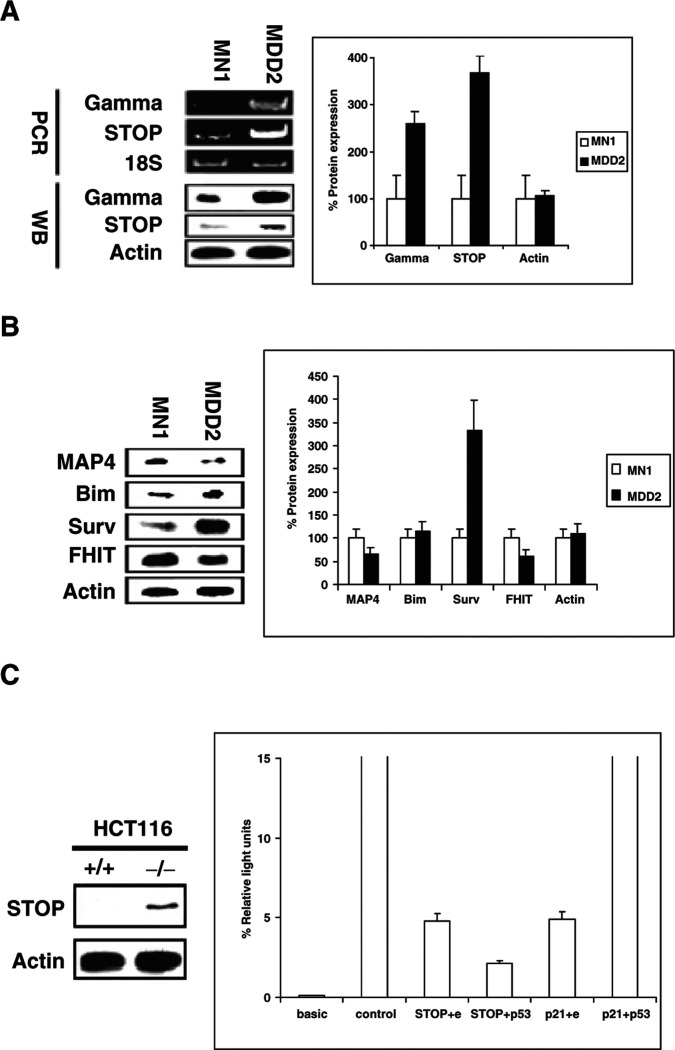
(**A**) Rt–PCR and Western blot analysis of *γ*-tubulin and STOP in MN1 (wt-p53) and MDD2 cells (mut-p53); (**B**) Western blot analysis of proteins interacting with microtubules; (**C**) Western blot analysis of STOP protein in HCT116 p53+/+ and p53−/− cells and negative regulation of the STOP promoter by wt-p53 in HCT1116 −/− colorectal cells. (A) and (B) represent one of the three experiments performed. Surv: survivin; Actin: *β*-actin. For (C), HCT116 p53-null colorectal cancer cells were cotransfected with the pGL-3-STOP-luciferase plasmid and pCMV-wt-p53 plasmid. Basic, pGL-3 basic plasmid; control, pGL-3 control plasmid; STOP+e, cotransfection of pGL-3-STOP-luciferase plasmid and empty pCMV; STOP+p53, cotransfection of pGL-3-STOP-luciferase plasmid and pCMV-wt-p53.

We then analysed the content of centriolar and centromeric proteins. Mut-p53 cells expressed more *γ*-tubulin mRNA and protein than MN-1 cells (Figure 3A

), indicating centrosome amplification in MDD2 cells. This is in accordance with our previous studies demonstrating that MDD2 cells, but not MN-1 cells, were tetraploid (DI=1.93) (Galmarini *et al*, 2001).

### Analysis of microtubule-associated proteins at basal condition

In the mut-p53 cells at basal conditions, STOP mRNA and protein were more abundant (Figure 3A), whereas MAP4 protein was decreased slightly (Figure 3B). Tau protein, MAP1b, and MAP2 were not expressed in either cell line (data not shown). Expression of the tumour suppressor protein FHIT (which binds tubulin) (Chaudhuri *et al*, 1999) was reduced in mut-p53 cells, whereas expression of the mitotic microtubule-associated antiapoptotic protein survivin (Li *et al*, 1998) was greatly increased (Figure 3B). Bim, a Bcl-2 family member reported to interact with microtubules (Strasser *et al*, 2000), was expressed at similar levels in both the cell lines (Figure 3B).

### p53 inactivates the transcription of the STOP gene in reporter gene assays

As shown in Figure 3C, STOP protein was expressed higher in HCT116 p53−/− than in HCT116 p53+/+ cells. Luciferase assays showed that STOP promoter activity was inhibited by 55% by cotransfection of the pGL-3-STOP-luc with wt-p53 (pCMV-wt-p53) in HCT116 p53 null cells in comparison with the control plasmid (pGL3 control) (Figure 3C). HCT1116 p53−/− cells cotransfected with the pGL-3-p21/Waf1-luc plasmid and the pCMV-wt-p53 plasmid showed an increase of 38% of p21/Waf1 promoter activity (data not shown).

## DISCUSSION

Our results show that dynamic instability of microtubules of mut-p53 cells was significantly increased as compared with that of their parental wt-p53 counterparts. The microtubule catastrophe frequency per minute was increased significantly, and microtubules spent more time in growing and shortening. They spent less time in pause and the pauses were shorter in duration. Their overall dynamics (dynamicity) were increased by 33%. However, the increase in overall dynamics in mut-p53 cells is only part of the picture, as in these cells the shortening rate was significantly decreased and the rescue frequency per length shortened was increased. As a result, the mean shortening length of the microtubules was reduced by 33%. The net result was that mut-p53 microtubules underwent frequent short excursions of shortening, but they lacked extensive changes in length as observed in wt-p53 microtubules.

Complex alterations in microtubule dynamics such as those which we have observed in mut-p53 cells may be involved in mechanisms of resistance to microtubule-targeted drugs. Recently published data showed that excessively rapid microtubule dynamics as well as suppressed dynamics were associated with resistance to paclitaxel in a human lung cancer line (Goncalves *et al*, 2001). One possibility is that different microtubules in the mitotic spindle, in particular, microtubules bound to kinetochores and interpolar microtubules, differ in terms of dynamic requirements. The complexity of the mitotic spindle requires fine tuning of the dynamics of all microtubules for proper function.

Mut-p53 cells also displayed a variety of alterations in microtubule protein composition that may be involved in the changes in microtubule dynamics. Although total tubulin content was slightly decreased in mut-p53 cells, the fraction of polymerised tubulin was higher. This change may result from the altered dynamics, from differences in microtubule stabilising protein content or function, or from alterations in some other undetermined factor regulating tubulin polymerisation. Post-translational alterations in *α*-tubulin, such as acetylation and detyrosination, have been preferentially found in microtubules with long half-lives, but it is not clear whether these modifications directly increase the lifespan of microtubules or simply accumulate as a result of prolonged existence of microtubule structures. The latter hypothesis would be compatible with the larger degree of acetylation and detyrosination observed in mut-p53 cells, since the microtubules in these cells undergo reduced shortening events, and thus are longer-lived than those in wt-p53 cells.

We also observed that the relative proportion of Class IV was greatly increased (seven-fold), whereas the proportions of classes I and II were reduced in mut-p53 cells. The *β*-tubulin isotype composition of microtubules *in vitro* can alter their dynamic properties (Panda *et al*, 1994; Derry *et al*, 1997), suggesting that the differences we observed in isotype composition between wt-p53 and mut-p53 cells may contribute to the observed differences in dynamics. These alterations may also be involved in drug resistance to microtubule-targeted drugs (Burkhart *et al*, 2001). The greatly increased expression of Class IV *β*-tubulin we observed would be predicted to result in increased resistance to paclitaxel (Derry *et al*, 1997), just as we observed for the mut-p53 cells.

The stabilising protein STOP and its corresponding mRNA were found to be much more highly expressed in the mut-p53 cells than in the wt-p53 cells, suggesting negative transcriptional regulation of STOP by p53 protein. This negative regulation was confirmed by a luciferase assay in HCT116 p53-null cells. The effects of STOP on microtubule dynamic instability are not known. However, STOP is an important factor in determining resistance of microtubules to cold-induced depolymerisation (Guillaud *et al*, 1998), and thus it may affect microtubule dynamics and could potentially contribute to higher polymerised tubulin content and/or to the reduced rate and extent of MT depolymerisation in mut p53 cells.

Microtuble-associated protein 4 is often assumed to stabilise microtubules. Other studies have found that expression of p53 downregulates expression of MAP4 (Murphy *et al*, 1996; Zhang *et al*, 1998). In contrast, we observed reduced expression of MAP4 in the mut-p53 cells, in conjunction with increased dynamic instability. Thus, our results are consistent with the predicted microtubule-stabilising capacity for MAP4. Although there is insufficient information available to predict the effects of changes in MAPs on microtubule dynamics with confidence, the fact that several MAPs that potentially affect microtubule stability are transcriptionally regulated by p53 strongly supports the role of p53 in regulating microtubule dynamics.

Survivin, an inhibitor of apoptosis associated with microtubules of the mitotic spindles, was found to be much more highly expressed in the mut-p53 cells than in the wt-p53 cells. Both overexpression of survivin and loss of wt-p53 expression have been detected in many tumours and this was associated with drug resistance (Altieri, 2001; Mirza *et al*, 2002). Overexpression of survivin influences microtubule dynamics and stabilisation of microtubules by directly regulating growth/catastrophe rates or via recruitment of MAPs or motor proteins participating in spindle dynamics (Giodini *et al*, 2002). In this context, overexpression of survivin related to the presence of mut-p53 cells may promote resistance to microtubule-targeted drugs by influencing microtubule dynamics and by inhibiting apoptosis.

Although we have previously reported that chemoresistance present in *p53*-deficient cells might be attributed to defective apoptotic pathways (Galmarini *et al*, 2001), alterations in microtubule composition and dynamics observed in mut-p53 cells might also contribute to the resistant phenotype. Our data indicate that p53 protein is involved in the structural and functional integrity of the microtubular cytoskeleton in complex and diverse ways. However, we cannot exclude that alterations in microtubular network observed in MDD2 cells were also because of genetic drift over time; chromosomal aberrations were present in the mut-p53 as demonstrated by the tetraploidy (Galmarini *et al*, 2001) and centrosome amplification observed in these cells. Different studies have demonstrated that the loss of p53 function leads to genetic instability allowing cells to accumulate mutations in many genes, including *β*-tubulin mutations (Giannakakou *et al*, 1997, 2000a). Silencing of p53 may thus predispose MDD2 cells to accumulate *β*-tubulin alterations that would alter microtubule composition and dynamics conferring resistance to microtuble-targeted drugs.

In summary, our results indicate that alterations in microtubule composition and dynamics observed in the presence of a dominant-negative p53 protein are likely to participate in microtubule-mediated mechanisms of resistance to microtubule-targeted drugs. This resistance may therefore result both from deficient apoptotic pathways and from modifications in microtubules and their associated proteins, thus reducing the sensitivity of intracellular targets to these compounds.
